# Intestinal Langerhans cell histiocytosis presenting with symptoms similar to inflammatory bowel disease: a case report

**DOI:** 10.3389/pore.2024.1611705

**Published:** 2024-03-28

**Authors:** Yuqing Liu, Zhenwei Chen, Lu Wang, Baizhou Li

**Affiliations:** Department of Pathology, The Fourth Affiliated Hospital of School of Medicine, and International School of Medicine, International Institutes of Medicine, Zhejiang University, Yiwu, Zhejiang, China

**Keywords:** Langerhans cell histiocytosis, histiocytosis, intestine, infant, case report

## Abstract

**Background:**

Langerhans cell histiocytosis is a rare disease characterized by the abnormal proliferation of Langerhans cells within a single organ or multiple organs. This case report aims to improve the knowledge of the presentation of gastrointestinal Langerhans cell histiocytosis to facilitate the diagnosis and management of this rare disorder.

**Case presentation:**

A 19-month-old female presented with repeatedly mucinous bloody stools. The abdominal ultrasound revealed a slightly enlarged spleen. The initial colonoscopy revealed chronic enteritis with a very early onset inflammatory bowel disease. After anti-inflammatory treatment without improvement, an intestinal biopsy was performed at The Forth Affiliated Hospital of Zhejiang University. The final intestinal biopsy and histopathology examination confirmed the presence of Langerhans cell histiocytosis. After diagnosis, additional lung and head imaging examinations revealed no abnormalities. Her condition improved gradually after being treated with chemotherapy (vincristine and prednisone) and molecular-targeted drug(dalafinil) treatment.

**Conclusion:**

The clinical symptoms of Langerhans cell histiocytosis involving the gastrointestinal tract are not specific and may resemble symptoms observed in inflammatory bowel disease and other primary gastrointestinal tumors. Therefore, in cases of infants presenting with inflammatory gastrointestinal symptoms that do not resolve after treatment, a biopsy is essential to obtain a differential diagnosis.

## Background

Histiocytosis is a group of rare diseases characterized by inflammation and accumulation of cells derived from monocytes and macrophages in different tissues. Langerhans cell histiocytosis (LCH) is the most common histiocytosis and occurs as a result of abnormal differentiation or proliferation of mononuclear phagocytic system cells [1]. LCH occurs mainly in girls below 2 years old. The disease has an incidence rate of about 5 per million [2]. LCH can develop in any location but tends to occur more frequently in bones, skin, pituitary, lungs, and lymph nodes. LCH rarely involves the digestive tract [3]. In this report, we describe a rare case of a 19-month-old female with LCH who presented with stubborn diarrhea and bloody stool.

## Case presentation

**FIGURE 1:**
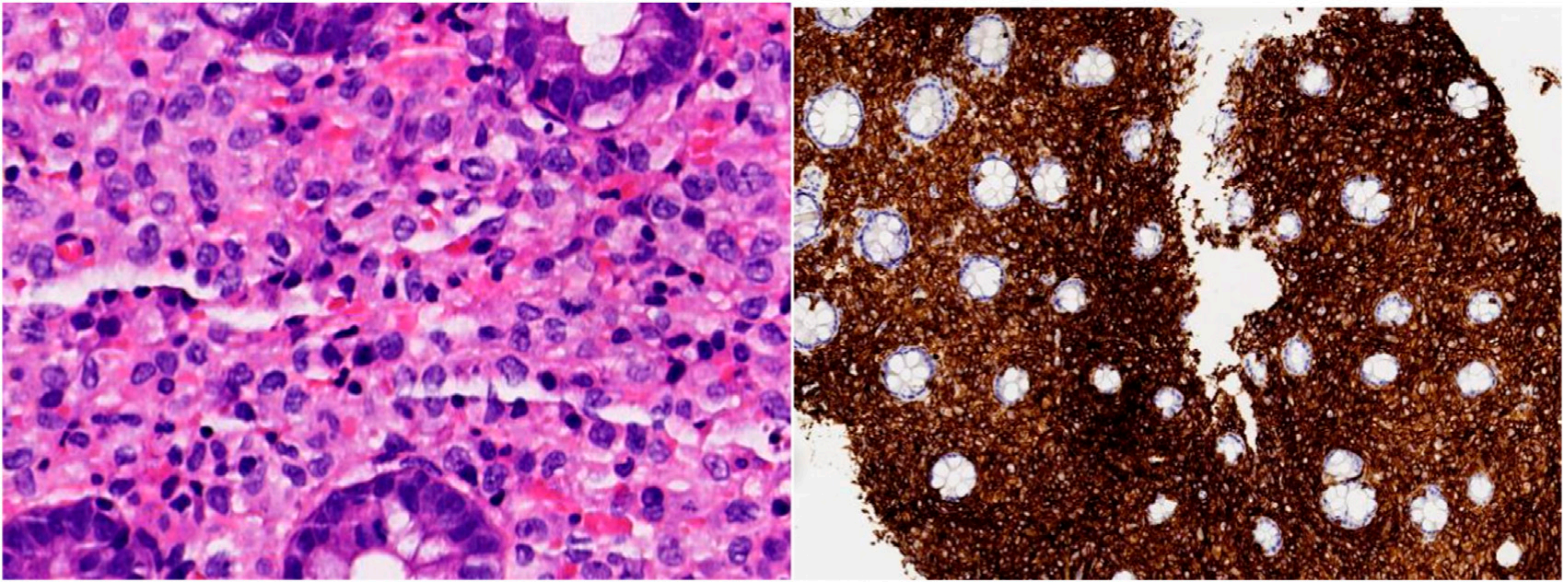
HE slides showing loose lamina propria strome within the ileocecal mucosa, evenly distributed powdery stained cells, and inflammatory eosinophil cell infiltration (Magnification ×400). IHC staining showing positive expression to CD1a in atypical cells (Magnification ×100).

**FIGURE 2:**
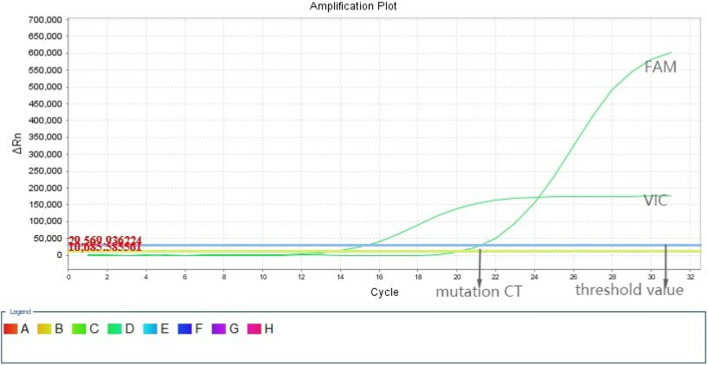
Fluorescence polymerase chain reaction amplification plot showing a *BRAF* gene mutation of the p.V600E type.The X-axis represents the cycle time (ct), and the Y-axis represents the fluorescence intensity.FAM and VIN are two fluorescent labels. A patient is deemed to have the BRAF p.V600E mutation if the amplification curve of the FAM signal is S-shaped and the mutation ct value is above 26.

A 19-month-old female presented with diarrhea for more than 10 months, 4 to 5 times a day, and no vomiting. The blood routine examination at the external hospital showed a decrease in hemoglobin, and a negative Coombs test. No abnormalities were detected in the bone marrow biopsy. An abdominal ultrasound revealed an enlarged spleen measuring 10 cm × 9 cm × 2 cm. No abnormalities were detected in the other organs. The patient was diagnosed with infectious diarrhea and treated with cefotaxime, imipenem, and vancomycin. Following the treatment, the diarrhea improved slightly. However, after some time the patient started to experience diarrhea with mucus and bloody stools, 4 to 5 times a day. A colonoscopy was performed which revealed proctitis. Pathological examination showed active inflammation of the rectal mucosa, accompanied by small vessel congestion and bleeding. The fecal test showed a positive result for Clostridioides difficile toxin. Based on these findings we thought that the patients might be suffering from infectious diarrhea, and did not consider the possibility that the patient may have an inflammatory bowel disease (IBD). Therefore the patient was treated with anti-inflammatories. Although, initially the patient experienced some improvements, the patient relapsed after some time. Two months later a second colonoscopy was performed which revealed similar changes. Nevertheless, the tissue samples obtained during the colonoscopy revealed chronic active inflammation in the colon, accompanied by focal granuloma formation. The two pathological biopsies confirmed the presence of inflammatory changes and no tumor cells were found. Therefore we suspected that the patient was suffering very early onset inflammatory bowel disease (VEO-IBD). Despite being treated with vancomycin, metronidazole, thalidomide, and prednisone, the therapeutic response remained unsatisfactory. The patient was admitted to our hospital on 21 August 2023. A clinical examination revealed normal hemoglobin levels, no skin abnormalities, and no lymph node swelling. However, the patient had an enlarged spleen and was underweight. The immediate family members of the child had no history of genetic diseases. A third colonoscopy was performed and showed diffuse congestion and edema of the total colon mucosa, erythema, and superficial ulcers. Therefore, multiple biopsies of the ileocecal region, transverse colon, descending colon, sigmoid colon, and rectum were obtained. The pathological results showed normal intestinal mucosa crypt structure with no significant reduction in the glandular structure of the mucosal lamina propria. These cells were evenly dispersed within the stroma and had an irregular nucleus and a pale, granular cytoplasm. Additionally, minor eosinophilic infiltration (approximately 10/high-power field) was observed in localized areas of the stroma. The immunohistochemical (IHC) results were positive for CD1a, S-100, CD68 and CD163. The Ki-67 marker showed a highly proliferative index. However, the IHC results for the CD30, ALK, CD117, CKpan, and CD138 tumor markers were negative (Figure 1). The *BRAF* p.V600E gene mutation was detected by fluorescence Polymerase Chain Reaction (PCR) using the Amplification Refractory Mutation System (ARMS) method (Figure 2). The immunohistochemical and BRAF p.V600E mutation results support the diagnosis of intestinal LCH. The computed tomography (CT) of the lungs and brain showed no abnormalities, except for LCH involvement of the intestine and spleen. Therefore, the girl was diagnosed with intestinal LCH with spleen involvement. After the diagnosis of this disease, the patient was treated with vincristine and prednisone chemotherapy drugs, as well as BRAF inhibitor dalafinil, for 2 months. The treatment significantly reduced the patient’s intestinal bleeding and diarrhea. An abdominal ultrasound revealed a reduction in the size of the spleen, indicating a gradual improvement in the patient’s condition.

## Discussion

The PubMed and Medbooks electronic databases were searched using the keywords “Langerhans cell histiocytosis,” “Digestive tract,” and “Intestine” to identify relevant research articles and case reports published in English between 1990 and 2023. The search strategy revealed 45 research papers. These papers included a total of 27 LCH cases involving the gastrointestinal tract in children under the age of 2 years. Table 1 summarizes the clinicalfeatures, treatment, and outcome of the 27 cases [4–24]. Majority of the cases were females, 21/27 (78%). Rash, diarrhea, vomiting and failure to thrive were the most common presenting features. Other presenting features were lungs, liver, spleen and bone involvement. Of these 27 cases, the outcome was not known in one case. Of the remaining 26 cases, 15 (58%) remissioned, 11 (42%) died.

**TABLE 1:** Summary of reported cases of LCH with gastrointestinal tract involvement.

Case	Refs	Age	Sex	Presenting symptoms	GI involvement	Other organ involvement	Treatment	Outcome
1	Wang et al. [4]	10 mo	F	Rash, diarrhea	colon	Skin	Vinblastin, prednisone	NAD
2	Wang et al. [4]	5 mo	F	Rash, diarrhea, hematochezia	ileocecal, descending colon, rectum	Skin, lung	Vinblastin, prednisone, dabrafenib	NAD
3	Zei et al. [5]	16 mo	F	Rash, diarrhea, vomiting	Duodenum, jejunum, colon	Skin, bone	Prednisolone, vinblastine	NAD
4	AbdullG-affar et al. [6]	11 mo	M	Rash, diarrhea, vomiting, edema, malnutrition	Colon	Skin, liver, spleen, gallbladder, lungs, ears	Prednisolone, vinblastin	PR
5	Hait et al. [7]	1 mo	F	Rash, rectal bleeding	Rectum, sigmoid colon	Skin, liver, spleen	Methotrexate, mercaptopurine, vinblastine, steroids	Died at 13 mo
6	Mayumi et al. [8]	9 mo	F	Rash, diarrhea, vomiting, fever, edema	Small intestine	Skin	Cytarabine, vincristine, prednisolone, 2-CdA	NAD
7	Shima et al. [9]	11 mo	F	Diarrhea, vomiting, edema	Colon	Skin, lung, liver, spleen	Vincristine, cytosine, arabinoside, prednisolone	CR
8	Levy et al. [10]	9 mo	F	Rash, edema, fever, otitis media	Stomach, duodenum, jejunum, colon	Skin, liver, mandible	Steroids, mercaptopurine	NAD
9	Godoy et al. [11]	5 mo	F	Rash, FTT, rectal bleeding	Rectum, sigmoid colon	Skin, liver, spleen	No treatment commented	No Commen-ted
10	Fang et al. [12]	16 mo	F	Rash, FTT, diarrhea	Stomach, duodenum, rectum	Skin	Vincristine, cytababine, prednisone	NAD
11	Miller et al. [13]	4 mo	F	Rash, diarrhea, fever, edem, vomiting	Duodenum, colon	Skin, liver	Vinblastine, prednisone	NAD
12	Choi et al. [14]	8 mo	M	Rash, fever, FTT,diarrhea, vomiting	Stomach, duodenum, colon	Skin, liver, spleen	Vinblastine, prednisone, 2-CdA	NAD
13	Lv et al. [15]	2 mo	M	Rash, diarrhea, hematochezia	Skin biopsy LCH+	Skin, ear, liver	Prednisone, vinblastine	Died at 3 mo
14	Boccon-Gibod et al. [16]	At birth	M	Rash, anemia, FTT	Duodenum, rectum	Skin, liver	Vinblastine, etoposide	Died at 17 wk
15	Boccon-Gibod et al. [16]	16 mo	F	Rash, fever, diarrhea	Stomach, duodenum, sigmoid colon	Skin, liver, spleen	Steroid, vinblastine, etoposide	CR
16	Gilmore et al. [17]	16 mo	F	Rash, fever, diarrhea, vomiting	Duodenum, colon	Skin, liver, spleen, skull, bone marrow	Prednisone, vinblastine, alpha interferon, solumedrol	CR
17	Santos-Machado et al. [18]	24 mo	F	Rash, diarrhea	No colonoscopy, Skin and bone marrow biopsy LCH+	Skin, liver, spleen, bone marrow	Vinblastine, methotrexate, prednisone, etoposide	Died at 26 mo
18	Santos-Machado et al. [18]	24 mo	M	Rash, jaundice, fever, pallor, diarrhea, loss of weight	No colonoscopy, palatal biopsy LCH+	Skin, liver	Prednisone, chlorambucil, hydroxyurea, cyclophosphamide	Died at 29 mo
19	Carlier-Mercier et al. [19]	At birth	F	Rash, vomiting, diarrhea	Rectum	Skin, liver, spleen, bone marrow	Steroids, vinblastine, cyarabine, etoposide, vincristine	Died at 32 mo
20	Patel et al. [20]	At birth	F	Rash, diarrhea	Jejunum	Skin, lung, bone	Unspecified chemotherapy	NAD
21	Geissma-nn et al. [21]	At birth	F	Rash, diarrhea, bloody stools	Colon, rectum	Skin, liver	Steroids, vinblastine, anti-CD1	Died at 3.5 mo
22	Geissma-nn et al. [21]	At birth	M	Rash, vomiting	Duodenum	Skin, liver	Steroids, vinblastine, etoposide, interferon-a	Died at 4 mo
23	Lee et al. [22]	At birth	F	Rash, bloody diarrhea	Rectum	Skin, bone, lung, liver, spleen	No treatment commented	Died at 16 wk
24	Lee et al. [22]	At birth	F	Rash, diarrhea	Rectum	Skin	Steroids, vinblastine, mercaptopurine, etoposide	Died at 10 mo
25	Yadav et al. [23]	22 mo	F	Rash, fever, vomiting, diarrhea	Duodenal, colon	Skin, liver, spleen, bone marrow	Vinblastine, prednisolone	CR
26	Yadav et al. [23]	17 mo	F	Fever, FTT, frequent loose stools	Duodenal, colon	Skin, liver, spleen, skull	Vinblastine, prednisolone, etoposide, cytarabine, 2-CdA	PR
27	Vetter-Laracy et al	At birth	F	Rash, diarrhea	Colon, rectum	Skin, lung, bone	Steroids, vinblastine, mercaptopurine	NAD

F, female; M, male; mo, month; wk, weeks; FTT, failure to thrive; 2-CdA, 2-Chlorodeoxyadenosine; NAD, non-active disease; CR, complete remission; PR, partial remission.

In this case study we reported on a 19-month-old female diagnosed with a rare case of LCH involving the entire colon. The 27 cases were found in the literature are similar to our case study. In the cases documented in the literature, we found that the intestinal symptoms of LCH patients with digestive tract involvement often mimic symptoms seen in other intestinal disorders. Colonoscopy can present polyp-like morphology, as well as display changes such as mucosal edema, superficial erosion, and bleeding ulcers. Due to the lack of specificity in the clinical features of the intestinal mucosa, a multi-point biopsy under colonoscopy guidance is often required to obtain a definitive diagnosis. Histologically, LCH presents as a mild to moderate decrease in glands in the lamina propria of the mucosa, with Langerhans cells distributed in patches or a diffuse pattern. Under high magnification, Langerhans cells exhibit a distinctive coffee bean-shaped convoluted nucleus with grooves and a pale, granular cytoplas. The stroma is usually mixed with a large number of eosinophils. The Langerhans cells express S100 protein, CD1a, langerin, and CD68. Ultrastructurally, Birbeck particles can be seen [25].

The colonoscopy of our patient revealed a diffuse distribution of erosion and ulcers throughout the entire colon. Among the five sites of the third intestinal biopsy, only the ileocecal, sigmoid, and rectal biopsies showed a mild reduction in the intrinsic glands of the lamina propria, and cells had the characteristic coffee bean-shaped nucleus of Langerhans cells evenly distributed in the lamina propria, while the transverse and descending colon biopsies did not show typical morphology. The histopathological analysis showed the characteristic nuclear groove of LCH and the IHC analysis was positive for the S-100, CD1a, and CD68 markers. These findings confirm that multiple biopsies are often required to diagnose LCH as the tissue sample obtained from a single-point biopsy may not have enough morphological changes to confirm the diagnosis.

The presence of abnormal Langerhans cells within the intestines could be used to differentiate LCH from other gastrointestinal disorders such as Ulcerative Colitis, Crohn’s Disease, Mastocytosis, and Anaplastic large-cell lymphoma. The ulcerative colitis lesions tend to be evenly distributed within the rectum and sigmoid colon. Under the microscope, lesions associated with ulcerative colitis typically display a twisted morphology accompanied by atrophic crypts. The mucosa surface of the large intestine often appears villous, with a notable infiltration of plasma cells within the mucosa. In contrast, Crohn’s disease predominantly affects the distal ileum. Lesions in Crohn’s disease are typically discontinuous and present a “cobblestone-like” appearance. Microscopic examination reveals patchy chronic inflammatory cell infiltration, irregular crypts, multiple segmental involvements, and the presence of Noncaseous granulomas. The digestive symptoms of IBD and LCH are very similar. Therefore, IHC staining for the S-100 protein, CD1a, and langerin markers is essential to obtain a differential diagnosis [26]. Mastocytosis mainly refers to the abnormal proliferation of mast cells within the mucosa. Mast cells can exhibit a series of forms, similar to histiocytes, and may also have obvious eosinophil infiltration. However, these cells express CD117 [27]. Anaplastic large-cell lymphoma also exhibits irregular nuclei and kidney-shaped cells. The lymphoid histiocyte pattern of anaplastic large-cell lymphoma also contains a large number of histiocytes. However, these cells express T cell, CD30, and ALK markers. Due to the unique immunohistochemical characteristics of LCH, immunohistochemistry is crucial for highlighting these subtle features and excluding other similar lesions. Additionally, our patient fecal test showed a positive result for Clostridioides difficile infection. This iatrogenic infection may have been precipitated by the administration of antibiotics used to treat the diarrhea before fecal examination.

LCH continues to be a subject of active research and exploration. Abnormal activation of the Mitogen-Activated Protein Kinase (MAPK) pathway was found in most LCH patients. About 60% of the MAPK pathway alterations in LCH have been attributed to the BRAF p.V600E gene mutations. As a result, BRAF inhibitors have been shown to have significant therapeutic effects on LCH patients with BRAF p.V600E gene mutations [28]. Our patient also carried the BRAF p.V600E mutation and responded well to molecular-targeted drug therapy with dalafinil.

LCH can involve single or multiple organs. The prognosis of LCH depends on the number of organ systems involved, the degree of involvement, and the age of the patient [29]. Yadav et al. reported that patients with multiple-system LCH have a worse mortality rate than those with single-system LCH (40% versus 7%). However, patients with gastrointestinal LCH are classified as having multi-system disease and tend to have a worse prognosis with a mortality rate of 55.5%. If other major organs such as the liver, spleen, and bone marrow are involved, the mortality rate increases to 78.5%. Therefore, when detecting LCH in the gastrointestinal tract, it is necessary to rule out the presence of other system involvement. At this point, supplementary imaging examinations including abdominal ultrasound, chest X-ray, and skeletal X-rays are essential to exclude multiple system involvement. If the patient presents with bicytopenia, pancytopenia, or persistent unexplained single cytopenia, a bone marrow biopsy should be performed [30]. The majority (93%) of LCH patients with digestive tract involvement experience skin lesions, and among these patients, over 80% prioritize presenting gastrointestinal symptoms, followed by skin lesions [31]. The appearance of a rash has important implications for the diagnosis of LCH. The 27 patients in Table 1 were all accompanied by skin lesions, however, our patient did not present with a rash that could indicate LCH. In addition, our patient did not have any osteolytic changes or lung lesions. At the beginning of the disease, the patient only presented with gastrointestinal symptoms and did not have the typical features of LCH such as rash and bone involvement. However, the patient had mild splenomegaly. For LCH with multiple system involvement, it is recommended to use first-line chemotherapy regimens of vincristine and prednisone. Although the prognosis of patients with LCH and gastrointestinal disorders tends to be poor, our patient responded well to chemotherapy and BRAF inhibitors. After 2 months of treatment, the condition of the patient gradually improved. We will continue to monitor the patient and the treatment will be adjusted according to the condition of the patient.

## Conclusion

The clinical manifestation of gastrointestinal LCH in children is not specific, thus making it difficult to distinguish it from other gastrointestinal diseases. Therefore, for young patients who present with gastrointestinal symptoms that do not respond to conventional treatment, multiple biopsies under colonoscopy guidance with IHC and molecular testing should be performed to exclude LCH. In addition, since LCH can affect multiple organs, other symptoms such as skin rash, spleen enlargement, and the presence of osteolytic or lung lesions should also be monitored as they could also indicate LCH. We believe that the findings of this case report could improve the differential diagnosis and management of LCH in young children with gastrointestinal symptoms.

## Data Availability

The original contributions presented in the study are included in the article/supplementary material, further inquiries can be directed to the corresponding author.

## Abbreviations

LCH, Langerhans cell histiocytosis; IBD, inflammatory bowel disease; VEO-IBD, very early onset inflammatory bowel disease; CT, Computed Tomography; IHC, immunohistochemical; PCR, polymerase chain reaction; ARMS, Amplification Refractory Mutation System; ct, cycle time; MAPK, Mitogen-Activated Protein Kinase; GI, gastrointestinal.
